# Influence of Abdominal Obesity on the Lipid-Lipoprotein Profile in Apoprotein E2/4 Carriers: The Effect of an Apparent Duality

**DOI:** 10.1155/2015/742408

**Published:** 2015-10-28

**Authors:** Sylvia Villeneuve, Diane Brisson, Daniel Gaudet

**Affiliations:** ^1^Department of Medicine, Université de Montréal, Montreal, QC, Canada H3T 1J4; ^2^ECOGENE-21, Chicoutimi, QC, Canada G7H 7K9; ^3^Douglas Mental Health University Institute, Research Centre, Verdun, QC, Canada H4H 1R3; ^4^Department of Psychiatry, McGill University, Montreal, QC, Canada H3A 1A1

## Abstract

*Background*. Apolipoprotein (Apo) E plays a key role in the handling of lipoprotein particles with ApoE2 and ApoE4 frequently having opposite effects compared to ApoE3. Some individuals simultaneously carry both E2 and E4 alleles. The impact of the ApoE2/4 genotype on lipid concentrations and its consequences on health remain poorly documented. *Objective*. This study compared the lipid profile between ApoE2/4 carriers and other ApoE genotypes in relation to the waist circumference. *Methods*. Cholesterol, triglyceride (TG), and ApoB concentrations were measured among 2,680 Caucasians. Multivariate logistic regression models were used to estimate the contribution of ApoE2/4 to various dyslipidemic profiles associated with abdominal obesity. *Results*. In presence of abdominal obesity, the lipid profile was as deteriorated in ApoE2/4 carriers as in carriers of other ApoE genotypes. There was a more pronounced effect on TG-rich lipoproteins, particularly in ApoE2/2 (a feature of type III dysbetalipoproteinemia), and non-high-density lipoprotein (HDL) cholesterol in ApoE4/4. Compared to ApoE2/2, ApoE2/4 carriers presented lower very-low-density lipoprotein (VLDL) cholesterol concentrations and VLDL-cholesterol/TG ratios, with or without obesity, and higher low-density lipoprotein (LDL) cholesterol concentrations. *Conclusion*. In presence of abdominal obesity, the influence of the ApoE2 allele could be less pronounced than that of ApoE4 among ApoE2/4 individuals.

## 1. Introduction

In addition to being a well-documented dyslipidemic factor by itself, abdominal obesity is known to influence the effect of lipid-related gene variants. Apolipoprotein (Apo) E is one of the most widely studied genes in relation to the lipid-lipoprotein metabolism. ApoE is a protein known for its key roles in the transport of cholesterol and other lipids in plasma and the central nervous system. The ApoE genetic polymorphism contributes to variations in plasma lipid-lipoprotein concentrations in normal and dyslipidemic populations [1]. ApoE includes three main alleles (*ε*2, *ε*3, and *ε*4) that give rise to three common isoforms (E2, E3, and E4) and six possible genotypes of which three are homozygous (ApoE2/2, ApoE3/3, and ApoE4/4) and three heterozygous (ApoE2/3, ApoE2/4, and ApoE3/4). With a frequency of around 80%, ApoE3 is the most common isoform, followed by ApoE4 (12%) and ApoE2 (8%) [2–4]. Accordingly, ApoE2/2 and ApoE2/4 carriers are less frequent and represent less than 3% of the population [3].

ApoE was discovered in 1973 by V. G. Shore and B. Shore [4]. Since then, its role in lipid/lipoprotein metabolism has been extensively investigated. Considerable advances have therefore been made in understanding the structure of ApoE and the impact of the three common isoforms on the lipid/lipoprotein metabolism, health status, and risk of disease. The ApoE isoforms differ from each other at one or two amino acid residues. ApoE3 contains cysteine at position 112 and arginine at position 158, while ApoE2 shows cysteine at both sites and ApoE4 contains arginine at both sites [1, 5]. However, their disparities are not limited to this molecular difference (Table 1). ApoE4 forms a unique salt bridge between Arg-61 in the N terminal and Glu-255 in the carboxy-terminal domain and a molten globule state that could reduce its stability, whereas in ApoE2, Cys-158 disrupts the salt bridge. The protein structure and lipid receptor binding of ApoE2 and ApoE4 are therefore differentially affected. While ApoE3 and ApoE4 have a normal low-density lipoprotein (LDL) receptor binding affinity, the binding of ApoE2 is defective [6]. Furthermore, ApoE3 and ApoE2 display a preference for high-density lipoproteins (HDL), whereas ApoE4 prefers LDL and very-low-density lipoproteins (VLDL) [7–9]. Accordingly, ApoE4 is frequently associated with increased plasma total and LDL-cholesterol levels in the homozygous and heterozygous (E3/E4) states. In contrast, ApoE2 is usually associated with normal or low lipid values in both homozygous and heterozygous (E2/E3) subjects. Finally, the ApoE2 isoform has been associated with higher triglyceride (TG) levels and lower ApoB levels than the ApoE4 isoform [10].

Together, these data indicate that ApoE influences the lipid/lipoprotein metabolism levels with ApoE2 and ApoE4 isoforms having opposite effects. Therefore, one could expect that on average ApoE2/4 carriers would present a phenotype similar to that of ApoE3/3, just as the mix of an acid and a base results in a neutral solution. But do they present such a phenotype? The answer to this question is uncertain, considering that the ApoE2/4 genotype has almost never been studied. Indeed, most of the previous studies of lipid metabolism have excluded ApoE2/4 carriers. In other studies, the strategy was to group them with ApoE2 carriers (homozygous and/or heterozygous) or ApoE4 carriers (homozygous and/or heterozygous). These strategies may have been justified and/or required by the small number of ApoE2/4 carriers. However, no validated scientific rationale appears to support these decisions. Moreover, considering that the modulator effect of adiposity would be stronger in ApoE2 carriers [14], could abdominal obesity significantly influence the ApoE2/4 carriers' lipid profile?

Due to a founder effect, the frequency of the ApoE2 isoform in the Saguenay-Lac-Saint-Jean (SLSJ) region (Québec, Canada) is among the highest ever reported [15]. Consequently, the ApoE2/4 genotype is not rare in this population, offering the appealing opportunity to answer these questions.

This paper therefore aims to compare the lipid-lipoprotein profile between ApoE2/4 carriers and other ApoE genotypes, in relation to waist circumference.

## 2. Material and Methods

### 2.1. Subjects and Clinical Data

The present study included 2,680 subjects (1,356 men and 1,324 women) from the SLSJ region of Quebec, Canada. All subjects were screened at the Chicoutimi Hospital Lipid Clinic or ECOGENE-21 Clinical Research Center and agreed to participate in studies on genetic determinants of type 2 diabetes (T2D) or coronary artery disease (CAD) combining genome wide scans and candidate gene strategies. They were selected on the basis of having a positive family or personal history of dyslipidemia, CAD, or T2D. Subjects with or suspected to have familial hypercholesterolemia (OMIM: 143890), those homozygous for a null LPL gene mutation or with plasma TG levels superior to 20 mmol/L (OMIM: 238600 and 144650), and those with a body mass index (BMI) greater than 40, taking drugs known to affect blood lipid levels, or with chronic alcohol consumption were excluded. The project was approved by the Chicoutimi Hospital Ethics Committee and written informed consent was obtained from each patient, in accordance with the Declaration of Helsinki.

Anthropometric variables were measured according to the recommendations from the Airlie Conference [16]. Subjects with an elevated waist circumference (>90 cm in men or 85 cm in women) were considered obese. Smoking habits were classified as never smoked versus ever smoked. T2D was defined according to the World Health Organization criteria as a 2-hour glucose concentration > 11.1 mmol/L following a 75 g oral glucose load [17]. CAD was defined by evidence of clinically documented myocardial infarction or angiographic analyses of coronary lesions [18, 19].

### 2.2. Biochemical Analysis

Blood samples were obtained in the morning after a 12-hour overnight fast from the antecubital vein into Vacutainer tubes containing EDTA. Cholesterol, TG, and glucose plasma levels were enzymatically measured on a CX7 Analyser (Beckman). Total cholesterol was determined in plasma and HDL after precipitation of VLDL and LDL (*d* > 1.006 g/mL) in the infranatant with dextran sulphate and magnesium chloride (MgCl_2_). In this case, plasma LDL-cholesterol levels were estimated using the Friedewald formula [20]. When TG levels were higher than 4.5 mmol/L, plasma LDL-cholesterol levels were calculated using a validated method [21]. Cholesterol and TG contents of VLDL, LDL, and HDL particles were also measured in a subsample of 1,531 subjects (742 men and 789 women, mean age = 49.2 ± 11.9) after ultracentrifugation of plasma. This subsample was comparable to the main sample in regard to age, gender, and ApoE distribution (all *p* > 0.05). Non-HDL-cholesterol concentrations were calculated by subtracting the HDL-cholesterol level from that of total plasma cholesterol. Total ApoB levels were determined using nephelometry. Clinical cutoff points used for plasma lipid-lipoprotein levels were established based on the primary prevention therapeutic target values of the National Cholesterol Education Program-Adult Treatment Panel (NCEP-ATP) III and the Canadian consensus for dyslipidemias [22, 23].

### 2.3. Genotyping

ApoE genotyping was performed using a restriction fragment length polymorphism (RFLP) analysis, with the Hha I restriction enzyme, as previously described [24]. Briefly, after cleavage of amplified sequences in specific regions, the DNA fragments were separated by electrophoresis on a polyacrylamide gel.

### 2.4. Statistical Analysis

Categorical variables were compared using the Pearson *χ* ^2^ statistic, which was also used to assess the Hardy-Weinberg equilibrium for the ApoE genotype distribution. Group differences for continuous variables were examined with a univariate analysis of variance (ANOVA) followed by the Bonferroni* post hoc* test or nonparametric Kruskal-Wallis tests followed by Mann-Whitney* U* tests when the homogeneity of the variance was not respected. Data that were not normally distributed were transformed using a log_10_ transformation and geometric means are shown in tables. The Bonferroni correction for multiple testing was applied. The independent sample Student's two-tailed* t*-test was performed to assess differences among obese and nonobese subjects within the same genotype. Binary logistic regression models were built in order to estimate the relative odds of dyslipidemias associated with the various ApoE genotypes including age, sex, waist circumference, and diabetes as covariates. The statistical significance level was set at *p* < 0.05. All statistical analyses were performed using the SPSS package (version 17.0, SPPS, Chicago, IL, USA).

## 3. Results

The observed frequencies of the ApoE genotypes did not differ from the expected frequencies according to the Hardy-Weinberg equilibrium (*p* > 0.05). As seen in Table 2, groups were comparable for age, sex, waist circumference, BMI, T2D, CAD, and hypertension expression as well as HDL-cholesterol levels. Though not significant, waist circumference and BMI tended to differ among groups. Heterozygous ApoE2/4 subjects had a lower level of TG than ApoE2/2 carriers but higher than ApoE3/3 carriers. They also had a higher total ApoB level than ApoE2/2.

**Figure 1:**
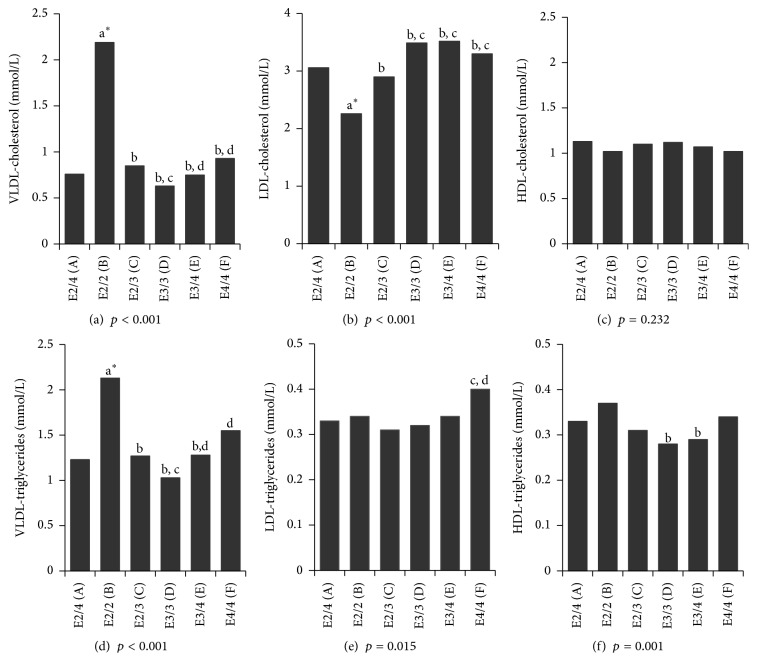
Lipid-lipoprotein profile determined after ultracentrifugation, according to the apolipoprotein E genotype, among a subsample of 1,531 subjects. Note: HDL, high-density lipoprotein; LDL, low-density lipoprotein; VLDL, very-low-density lipoprotein. Significantly different (*p* < 0.05) as compared to ^a^**∗**^^E2/4, to ^b^E2/2, to ^c^E2/3, to ^d^E3/3, and to E3/4 and when corrected for multiple testing. All *p* values concerning VLDL-cholesterol, VLDL-triglycerides, and LDL-triglycerides concentrations were obtained after log_10_ transformation of the data, and geometric means are shown. Men/women: E2/4 (36/36); E2/2 (37/37); E2/3 (197/199); E3/3 (324/356); E3/4 (129/143); E4/4 (19/18).

Figure 1 presents the lipid-lipoprotein profile following ultracentrifugation among a subsample of 1,531 subjects according to the ApoE genotype. Looking at significant group differences concerning the ApoE2/4 genotype, the latter had higher LDL-cholesterol but lower VLDL-cholesterol and VLDL-TG concentrations than ApoE2/2 carriers.

**Figure 2:**
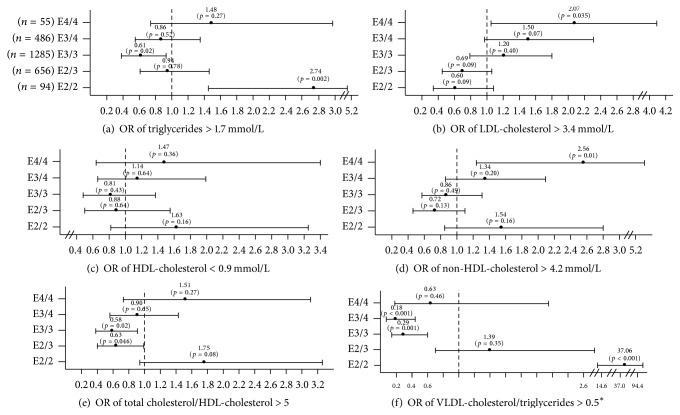
Estimated relative risk (odds ratio, OR) of hyperlipidemia associated with the apolipoprotein E genotype, among a subsample of 1,531 subjects. The ApoE2/4 genotype was considered as the reference group to which an odds ratio of one was set for comparison purposes. Odds ratio ± 95% CI. Covariates included in mode l are age, sex, type 2 diabetes, and BMI.

Figure 2 shows the risk (odds ratio) of exhibiting hyperlipidemia when compared to ApoE2/4 carriers. Results showed that ApoE2/4 carriers were less likely than ApoE2/2 carriers, but more likely than ApoE3/3 carriers, to suffer from hyperTG. They were at a lower risk of having high LDL-cholesterol levels or high non-HDL-cholesterol levels than ApoE4/4 subjects. They showed an increased risk of having a high total cholesterol/HDL-cholesterol ratio when compared to ApoE2/3 and ApoE3/3 carriers. Finally, they had a lower risk of having an abnormally high VLDL-cholesterol/TG ratio than ApoE2/2 carriers but a higher risk than ApoE3/3 and ApoE3/4.

Table 3 displays the lipid-lipoprotein profile according to the ApoE genotype and abdominal obesity. In all genotypes, the level of HDL-cholesterol was lower and the levels of TG and VLDL-cholesterol were higher in individuals with abdominal obesity when compared to those without abdominal obesity. In ApoE2/4 carriers, the total cholesterol/HDL-cholesterol ratio and VLDL-TG were also higher in individuals with abdominal obesity when compared to those without abdominal obesity. Furthermore, our results showed that nonobese ApoE2/4 carriers had higher TG and VLDL-cholesterol levels than nonobese ApoE3/3 carriers, a higher LDL-cholesterol level than nonobese ApoE2/2 and ApoE2/3 carriers, and a higher non-HDL-cholesterol level than nonobese ApoE2/3, but a lower VLDL-cholesterol level than ApoE3/3 and a lower VLDL-cholesterol/TG ratio than nonobese ApoE2/2 carriers. ApoE2/4 carriers with abdominal obesity had a lower level of TG than obese ApoE2/2 carriers, a lower level of non-HDL-cholesterol than obese ApoE2/2 and obese ApoE4/4 carriers, a lower total cholesterol/HDL-cholesterol ratio than ApoE4/4 carriers, and finally a lower VLDL-cholesterol/TG ratio than ApoE2/2 carriers.

## 4. Discussion 

The ApoE polymorphism is a key modulator of the lipid/lipoprotein metabolism, with ApoE2 and ApoE4 having mainly opposite effects. Our results suggest that although both isoforms play a role in the lipid/lipoprotein metabolism in ApoE2/4 subjects, the influence of the ApoE2 allele is less pronounced than that of ApoE4, particularly in presence of abdominal obesity. This influence varies according to the lipid/lipoprotein subtype and presence of a secondary dyslipidemic factor. In nonobese subjects, the ApoE2/4 carriers have higher LDL- and non-HDL-cholesterol concentrations than ApoE2/3 carriers, closer to those of ApoE3/4 and ApoE4/4 carriers. In presence of abdominal obesity, the lipid-lipoprotein profile is as deteriorated in ApoE2/4 carriers as in carriers of other ApoE genotypes, with the exception of the TG-rich lipoprotein fractions that are higher in ApoE2/2 carriers. This is a feature of type III dysbetalipoproteinemia, a rare disorder mainly associated with the ApoE2 isoform. The other exception is that non-HDL-cholesterol and total cholesterol/HDL-cholesterol ratio are higher in obese ApoE4/4 [25, 26].

Total TG concentrations among ApoE2/4 subjects were higher than those found in ApoE3/3 carriers. This result is characteristic of the ApoE2 isoform, which clears TG-rich particles from the plasma more slowly than ApoE3 and ApoE4 [27]. Importantly, the TG levels found in ApoE2/4 carriers were not as high as those found in ApoE2/2 carriers, suggesting that the ApoE4 isoform also, to a lesser amplitude, influences the TG metabolism in ApoE2/4 carriers. The present study also showed a trend for an increasing gradient of total ApoB concentrations ranging from ApoE2/2 carriers to ApoE4/4 carriers; this result replicates previous work [28]. Results obtained after the ultracentrifugation showed the same trend for LDL-cholesterol, placing ApoE2/4 closer to ApoE3/4 than ApoE2/3. In this last finding, ApoE2/2 carriers presented the lowest LDL-cholesterol concentration, which is in accordance with the *ε*2 allele binding deficiency to LDL receptors [1, 29]. Those findings are consistent with previous work reporting lower LDL-cholesterol levels in ApoE2 carriers than ApoE4 carriers [11, 29, 30]. Results obtained after the ultracentrifugation also showed that ApoE2/4 carriers have lower VLDL-cholesterol and VLDL-TG concentrations than ApoE2/2 carriers, suggesting that the ApoE4 isoform influences the clearance of VLDL particles in ApoE2/4 carriers. Altogether, these results imply that ApoE2 and ApoE4 isoforms both influence the ApoE2/4 lipid/lipoprotein metabolism. Supporting this idea, the findings of Ikewaki and collaborators [31] suggest that ApoE2 and ApoE4 have distinct metabolic pathways in heterozygous ApoE2/4 individuals.

As mentioned previously, ApoE2/4 carriers had higher TG levels than ApoE3/3 carriers. Bringing this observation further, we found that ApoE2/4 subjects had a higher risk of hypertriglyceridemia than ApoE3/3 carriers, but less risk than ApoE2/2 carriers. Furthermore, ApoE2/4 subjects were at decreased risk of LDL hypercholesterolemia and high non-HDL-cholesterol when compared to ApoE4/4 carriers. Compared to ApoE2/3 and ApoE3/3, they showed an increased risk of having a total cholesterol/HDL-cholesterol ratio above 5, a ratio often used as a marker of CAD risk [32]. Finally, ApoE2/4 carriers were at a lower risk of exhibiting a VLDL-cholesterol/TG ratio greater than 0.5 when compared to ApoE2/2 but at higher risk than ApoE3/3 and ApoE3/4 carriers. This last ratio is used for estimating the proatherogenic IDL subfraction levels and as a marker of type III dysbetalipoproteinemia risk [32]. Moreover, not surprisingly, most non-ApoE2/2 type III subjects were ApoE2 heterozygous carriers, around 15% of ApoE2/3 and ApoE2/4 subjects having a type III phenotype, whereas the prevalence of type III among ApoE3/3 and ApoE3/4 carriers was below 5%. Altogether, those results suggest that ApoE2/4 is protective against hyperlipidemia when compared to ApoE2/2 and ApoE4/4. However, when compared to ApoE3/3, the latter have a small increased risk for hyperTG and hyperIDL. They are also at increased odds of having a cholesterol/HDL-cholesterol ratio above 5.

Additional primary or secondary factors, some very common such as obesity, could influence those risks. Our results suggest that abdominal obesity, defined by an elevated waist circumference (>90 cm in men or 85 cm in women), affects HDL and TG concentrations as well as the cholesterol/HDL-cholesterol ratio in ApoE2/4 carriers similar to what is observed for other ApoE genotypes. More specifically, we found that obese subjects were characterized by lower concentrations of HDL-cholesterol than nonobese subjects. Supporting our results, Srinivasan and collaborators (2001) found that both obese ApoE2 (E2/2 and E2/3) and ApoE4 (E3/4 and E4/4) carriers showed reduced HDL-cholesterol when compared to their respective nonobese groups [33]. Obese ApoE2/4 subjects also had increased TG concentrations, which is consistent with previous studies showing elevated TG levels and an increased hypertriglyceridemia risk in obese ApoE4 carriers [34, 35]. The cholesterol/HDL-cholesterol ratio showed the same pattern, with obese individuals having a higher ratio than nonobese individuals.

Potential limitations of our study include selection bias, considering that subjects were selected on the basis of having a positive family or personal history of dyslipidemia, CAD, or T2D. It could therefore be more difficult to observe significant differences for the expression of cardiometabolic risk covariates. Moreover, screening of subjects from a founder population has provided a certain ethnic homogeneity to our sample, which could also be considered as a bias. However, it is precisely these recruitment strategies that provided enough ApoE2/4 subjects to allow such a study. Until now, no study had included so many ApoE2/4 subjects. Now that these preliminary results have been obtained, they will need to be validated in larger samples and diversified populations to ensure their external validity. Considering the small prevalence of this genotype, meta-analyses would be an appealing strategy to determine if the current results are representative of the general population.

Results of our study were also in line with the literature as regards the impact of the different genotypes on the lipid/lipoprotein metabolism. Since other factors such as high-carbohydrate diets or certain chronic diseases have been reported to influence the lipid/lipoprotein metabolism, it would have been interesting to include them in this study. Further research should assess those factors. Finally, it would also be interesting to perform such analyses after a standardized meal. There are less postprandial studies than studies performed in the fasting state. We believe that the addition of postprandial and dynamic analyses could provide a more comprehensive understanding of the ApoE metabolism and maybe the key unlocking some mechanisms.

In summary, results of this study suggest that both ApoE2 and ApoE4 isoforms play a role in the lipid/lipoprotein metabolism of ApoE2/4 subjects and that the magnitude of their respective importance depends on the lipid/lipoprotein subtype. Furthermore, obesity is associated with an increased risk of dyslipidemia in ApoE2/4 carriers similar to what can be observed for other ApoE genotypes (Table 4). However, in presence of abdominal obesity, the influence of the ApoE2 allele appears less pronounced than that of ApoE4.

## 5. Conclusions

These data have provided new insight on the lipid/lipoprotein profile of ApoE2/4 carriers and their hyperlipidemia risk. This study did not however directly assess the risk of CAD in ApoE2/4 carriers. Given the influence of lipid concentrations on the risk of CAD, studies that would directly examine the CAD risk of ApoE2/4 would give novel insights on the role of ApoE in human health. Increasing our knowledge on the duality of ApoE4 and ApoE2 isoforms may also help create new treatments for a variety of diseases. Indeed, even if this paper focused on the impact of ApoE on lipid concentrations, ApoE is a well-known risk factor for many diseases including Alzheimer's disease. Finally, because the current study was performed in a founder population, these data need to be replicated with other, more diversified samples.

## Figures and Tables

**Table 1:** Main differences between ApoE2 and ApoE4 [10–13].

	ApoE2	ApoE4
Isoform difference	Cys-112 Cys-158	Arg-112 Arg-158

Structure difference	Cys-158 disrupts the salt bridge	Domain interaction (formation of a salt bridge between Arg-61and Glu-255)

Protein stability	High	Very low

Binding preferences	Preferentially binds to small phospholipid-rich HDL Severely defective in LDL receptor binding activity (<2% binding activity compared with ApoE3)	Binds preferentially to large, triglyceride-rich VLDL Binds avidly to LDL receptors

Serum lipid association	Tends to be associated with increased levels of ApoE and triglyceride and decreased levels of ApoB and cholesterol	Tends to be associated with decreased ApoE but increased ApoB and cholesterol levels


Arg: arginine; Cys: cysteine; HDL: high-density lipoprotein; LDL: low-density lipoprotein; VLDL: very-low-density lipoprotein.

**Table 2:** Subjects' characteristics according to the apolipoprotein E genotype.

	ApoE 2/4 (A)	ApoE 2/2 (B)	ApoE 2/3 (C)	ApoE 3/3 (D)	ApoE 3/4 (E)	ApoE 4/4 (F)	*p* value
Men/women	51/53	48/46	326/330	660/625	247/239	24/31	NS
Age (years)	48.8 ± 12.7	50.8 ± 13.07	50.3 ± 11.2	48.8 ± 12.1	48.8 ± 11.9	46.2 ± 11.3	NS
Waist circumference (cm)	88.2 ± 13.2	90.7 ± 12.3	90.5 ± 13.3	89.8 ± 13.9	88.5 ± 13.1	86.6 ± 12.6	0.056
BMI (kg/m^2^)	26.6 ± 4.3	27.3 ± 4.3	26.9 ± 4.6	26.6 ± 4.5	26.5 ± 4.4	25.8 ± 4.3	0.082
Smoking habits^1^ (%)	66.3	61.7	68.4	65.4	67.3	58.2	NS
Type 2 diabetes^1^ (%)	10.6	11.7	14.5	14.7	14.4	9.1	NS
Hypertension^1^ (%)	42.3	38.3	39.6	37.1	41.2	30.9	NS
CAD (%)	29.8	28.7	27.9	28.4	24.5	27.3	NS
Triglycerides (mmol/L)^*∗*,†^	2.06 ± 1.95	2.97 ± 3.26^a^	1.88 ± 2.02^b^	1.60 ± 1.99^a,b,c^	1.81 ± 2.18^b,d^	2.20 ± 3.46	<0.001
Total cholesterol (mmol/L)^†^	5.52 ± 1.34	6.50 ± 3.16	5.37 ± 1.35^b^	5.41 ± 1.05^b^	5.64 ± 1.18^b,c,d^	6.02 ± 1.11^c,d^	<0.001
LDL-cholesterol (mmol/L)^†^	3.17 ± 0.89	2.66 ± 1.08	2.96 ± 0.90	3.27 ± 0.83^b,c^	3.37 ± 0.82^b,c^	3.40 ± 0.85^b,c^	<0.001
HDL-cholesterol (mmol/L)	1.17 ± 0.44	1.13 ± 0.55	1.21 ± 0.44	1.22 ± 0.41	1.20 ± 0.42	1.14 ± 0.42	NS
Total ApoB (g/L)^§,†^	1.05 ± 0.24	0.83 ± 0.32^a^	1.00 ± 0.26^b^	1.06 ± 0.24^b,c^	1.12 ± 0.25^b,c,d^	1.20 ± 0.27^b,c,d^	<0.001

Data are mean ± S.D.

Note: ApoB: apolipoprotein B-100 measured on delipidated plasma; BMI: body mass index; CAD: coronary artery disease; HDL: high-density lipoprotein; LDL: low-density lipoprotein.

^1^Proportion (%) of individuals who ever smoked (smoking habits), are diabetic (type 2 diabetes), and are hypertensive.

^§^Among a subsample of 1,915 subjects.

Significantly different (*p* < 0.05) as compared to ^a^ **E**2/4; to ^b^E2/2; to ^c^E2/3; to ^d^E3/3.

^*∗*^ *p* value obtained after log_10_ transformation of the data; geometric means are shown.

^†^Kruskal-Wallis tests followed by Mann-Whitney *U* tests; geometric means are shown.

NS = *p* > 0.05.

**Table 3:** Lipid-lipoprotein profile according to apolipoprotein E genotype and abdominal obesity.

	ApoE 2/4 (A)	ApoE 2/2 (B)	ApoE 2/3 (C)	ApoE 3/3 (D)	ApoE 3/4 (E)	ApoE 4/4 (F)	ANOVAs or KW	ANOVAs or KW^*∗∗*^
Nonobese/obese	51/53	32/62	268/388	560/725	228/258	28/27	*p* value	*p* value
Triglycerides^1*∗*^								
Nonobese	*1.76 ± 1.65*	*2.24 ± 2.34*	*1.45 ± 1.75* ^b^	***1.26 ± 1.75*** ^a,b,c^	*1.46 ± 1.41* ^b,d^	*1.56 ± 1.11*	<0.001	<0.001
Obese	*2.41 ± 2.15*	***3.44 ± 3.54*** ^a^	*2.25 ± 2.11* ^b^	*1.93 ± 2.09* ^b,c^	*2.18 ± 2.60* ^b^	*3.14 ± 4.43* ^d^	<0.001	<0.001
LDL-cholesterol^1^								
Nonobese	3.32 ± 0.93	2.71 ± 1.09^a^	***2.86 ± 0.85*** ^a^	*3.27 ± 0.84* ^b,c^	3.45 ± 0.82^b,c^	3.53 ± 1.71^b,c^	<0.001	<0.001
Obese	3.28 ± 0.85	2.97 ± 1.07	*3.27 ± 0.90*	*3.48 ± 0.81* ^b,c^	3.51 ± 0.84^b,c^	3.56 ± 0.98^b^	<0.001	<0.001
HDL-cholesterol^1^								
Nonobese	*1.32 ± 0.51*	*1.39 ± 0.75*	*1.38 ± 0.49*	*1.38 ± 0.43*	*1.35 ± 0.44*	*1.39 ± 0.30*	NS	NS
Obese	*1.03 ± 0.29*	*1.00 ± 0.35*	*1.10 ± 0.36*	*1.10 ± 0.36*	*1.06 ± 0.36*	*0.88 ± 0.36* ^c,d^	0.006	<0.001
VLDL-cholesterol^1,§^								
Nonobese	*0.66 ± 1.17*	***1.41 ± 2.21*** ^a^	*0.60 ± 0.72* ^b^	***0.43 ± 0.60*** ^a,b,c^	*0.56 ± 0.76* ^b,d^	*0.67 ± 0.53*	<0.001	<0.001
Obese	*0.95 ± 0.70*	***2.28 ± 2.27*** ^a^	*1.01 ± 1.19* ^b^	*0.76 ± 0.71* ^b,c^	*0.88 ± 1.16* ^b^	*1.27 ± 1.45* ^b,d^	<0.001	<0.001
VLDL-triglycerides^1,§^								
Nonobese	*0.99 ± 1.32*	1.67 ± 2.64	*0.87 ± 1.19* ^b^	*0.68 ± 1.14* ^b^	*0.93 ± 1.38* ^b,d^	*1.05 ± 1.02*	<0.001	<0.001
Obese	*1.57 ± 1.62*	2.15 ± 1.87	*1.61 ± 1.94*	*1.31 ± 1.36* ^b,c^	*1.58 ± 2.56*	*2.23 ± 3.13* ^d^	<0.001	<0.001
Non-HDL-cholesterol^1,†^								
Nonobese	4.18 ± 1.60	*3.81 ± 3.01*	***3.54 ± 1.34*** ^a^	*3.83 ± 1.04* ^c^	*4.15 ± 1.17* ^c,d^	*4.28 ± 0.98* ^b^	<0.001	<0.001
Obese	4.44 ± 1.06	***5.80 ± 3.48*** ^a^	*4.52 ± 1.43* ^b^	*4.42 ± 1.10* ^b^	*4.62 ± 1.30* ^b^	***5.46 ± 1.25*** ^a,c,d,e^	<0.001	<0.001
Total cholesterol/HDL-cholesterol								
Nonobese	*4.50 ± 2.51*	4.70 ± 24.28	*3.81 ± 2.75*	*3.97 ± 1.62*	*4.33 ± 1.99* ^c,d^	*4.19 ± 1.38*	<0.001	<0.001
Obese	*5.54 ± 1.91*	7.42 ± 8.61	*5.45 ± 3.11* ^b^	*5.32 ± 4.53* ^b^	*5.71 ± 3.54* ^d^	***8.11 ± 13.52*** ^a,c,d,e^	<0.001	<0.001
VLDL-cholesterol/triglycerides^*∗*§,†^								
Nonobese	0.38 ± 0.15	***0.64 ± 0.24*** ^a^	*0.40 ± 0.10* ^b^	*0.33 ± 0.10* ^b,c^	*0.35 ± 0.08* ^b,c^	*0.38 ± 0.09* ^b^	<0.001	<0.001
Obese	0.43 ± 0.10	***0.78 ± 0.23*** ^a^	*0.44 ± 0.10* ^b^	*0.38 ± 0.08* ^b,c^	*0.38 ± 0.07* ^b,c^	*0.40 ± 0.07* ^b^	<0.001	<0.001

Data are mean ± SD and shown when no covariates are included in the models.

Note: HDL: high-density lipoprotein; LDL: low-density lipoprotein; non-HDL-cholesterol: cholesterol total − high-density lipoprotein cholesterol; VLDL: very-low-density lipoprotein; KW: Kruskal-Wallis tests.

^1^Units are in mmol/L.

^§^Among a subsample of 1,531 subjects.

Significantly different (*p* < 0.05) as compared to ^a^ **E**2/4; to ^b^E2/2; to ^c^E2/3; to ^d^E3/3; and to ^e^E3/4 within the same nonobese/obese group.

Significant differences between nonobese and obese subjects within the same genotype group are in italic font.

^*∗*^ *p* value obtained after log_10_ transformation of the data; geometric means are shown.

^†^Kruskal-Wallis tests followed by Mann-Whitney *U* tests; geometric means are shown.

^**∗∗**^When controlled for age, sex, and type 2 diabetes.

**Table 4:** Summary of the main findings.

ApoE2/4 lipid/lipoprotein level (or risk) compared to					
	ApoE 2/2	ApoE 2/3	ApoE 3/3	ApoE 3/4	ApoE 4/4
TG	Lower	—	Higher	—	—
Total C	—	—	—	—	—
LDL-C	Higher^1^	—	—	—	—
HDL-C	—	—	—	—	—
VLDL-C	Lower	—	—	—	—
VLDL-TG	Lower	—	—	—	—
LDL-TG	—	—	—	—	—
HDL-TG	—	—	—	—	—
Total ApoB	Higher	—	—	—	—
TG > 1.7 mmol/L	Lower	—	Higher	—	—
LDL-C > 3.4	—	—	Higher	—	Lower
HDL-C < 0.9 mmol/L	—	—	—	—	—
Non-HDL-C > 4.2 mmol/L	—	—	—	—	Lower
Total C/HDL-C. > 5	—	Higher	Higher	—	—
VLDL-C/TG > 0.5	Lower	—	Higher	Higher	—

Present is a summary of the lipid/lipoprotein differences between ApoE2/4 carriers and the other genotypes. Lower means that ApoE2/4 carriers present a lower level (or risk) than the selected genotype while higher means that ApoE2/4 carriers present a higher level (or risk) than the selected genotype. This summary is based on Table 2 and Figures 1 and 2.

Note: ApoB: apolipoprotein B-100 measured on delipidated plasma; C: cholesterol; HDL: high-density lipoprotein; LDL: low-density lipoprotein; TG: triglycerides

^1^The difference between ApoE2/2 and ApoE2/4 for LDL-C only reached significance when measured after ultracentrifugation.
